# Nanotherapeutics Overcoming the Blood-Brain Barrier for Glioblastoma Treatment

**DOI:** 10.3389/fphar.2021.786700

**Published:** 2021-11-25

**Authors:** Lin Tang, Yicheng Feng, Sai Gao, Qingchun Mu, Chaoyong Liu

**Affiliations:** ^1^ Beijing Advanced Innovation Center for Soft Matter Science and Engineering, Beijing University of Chemical Technology, Beijing, China; ^2^ College of Life Science and Technology, Beijing University of Chemical Technology, Beijing, China; ^3^ The People’s Hospital of Gaozhou, Gaozhou, China

**Keywords:** glioblastoma, blood-brain barrier, nanotherapeutics, drug delivery, nanocarriers

## Abstract

Glioblastoma (GBM) is the most common malignant primary brain tumor with a poor prognosis. The current standard treatment regimen represented by temozolomide/radiotherapy has an average survival time of 14.6 months, while the 5-year survival rate is still less than 5%. New therapeutics are still highly needed to improve the therapeutic outcome of GBM treatment. The blood-brain barrier (BBB) is the main barrier that prevents therapeutic drugs from reaching the brain. Nanotechnologies that enable drug delivery across the BBB hold great promise for the treatment of GBM. This review summarizes various drug delivery systems used to treat glioma and focuses on their approaches for overcoming the BBB to enhance the accumulation of small molecules, protein and gene drugs, etc. in the brain.

## Introduction

Primary brain tumor or spinal cord tumors are tumors that begin in the brain or spinal cord, brain tumor accounts for 85–90% of all primary tumors that occur on the central nervous system (CNS), which is the 10th main cause of death in the world (Ostrom et al., 2020). The most common malignant brain and other central nervous system tumors are glioblastoma (GBM) derived from astrocytes (14.5% of all tumors). Patients suffering from GBM have a median survival time of only 8 months. At present, the standard of treatment for newly diagnosed GBM is surgery, followed by radiation and oral chemotherapy, and the median survival time can be extended to 14 months. FDA has approved anti-glioma therapy drugs, including temozolomide (Esteller et al., 2000; Hegi et al., 2004), a DNA alkylation reagent, often used with radiotherapy; bevacizumab, a human monoclonal antibody that inhibits vascular endothelial growth factor (VEGF), and the combination with chemotherapy are related to the long-term survival rate of patients (Vredenburgh et al., 2007; Miller et al., 2008). Despite extensive efforts, the overall survival rate is not significantly changed over time (Delgado-Lopez and Corrales-Garcia, 2016). There is an urgent need to develop more innovative therapeutics to advance GBM management.

The treatment of GBM faces numerous challenges (Delgado-Lopez and Corrales-Garcia, 2016; Anjum et al., 2017). The diffuse and infiltration of tumors into healthy tissues limits the feasibility of surgery, also the prognosis of radiotherapy and chemotherapy is poor. GBM has a significant intertumoral and intratumoral genomic heterogeneity, some tumor cells may have good responses to specific therapeutics, while others may have no response at all. The rapid proliferation and drug resistance of tumor cells increase the difficulty of treatment. Most importantly, the blood-brain barrier (BBB), the protective barrier of the central nervous system, limits the delivery of drugs to the brain parenchyma and the sensitivity of the brain to therapeutic effects. Even with the progress of the tumor, the structure and function of BBB are changed after the rupture of the tumor membrane and the deterioration of the tumor. Eventually, BBB is replaced by the blood-brain tumor barrier (BBTB), which hinders the delivery of most anti-tumor drugs (Ningaraj et al., 2002; Zhan and Lu, 2012). About 98% of small molecules and almost all biological macromolecule drugs (such as growth factors, monoclonal antibodies, etc.) cannot enter the central nervous system to exert therapeutic effects (René and Parks, 2021). This directly led to the failure of many drugs in clinical trials.

**FIGURE 1:**
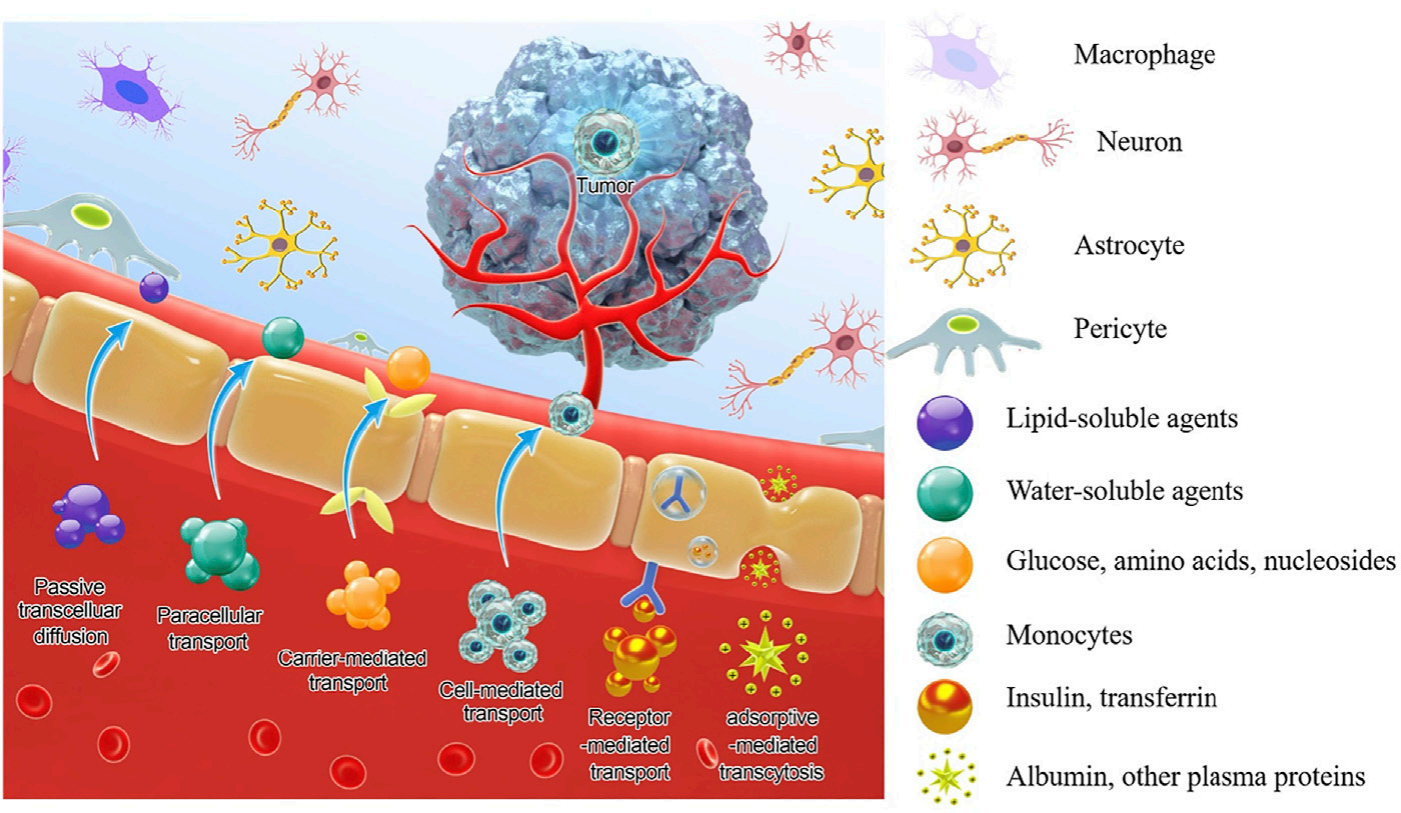
Structure of the BBB and pathways to cross the BBB.

**FIGURE 2:**
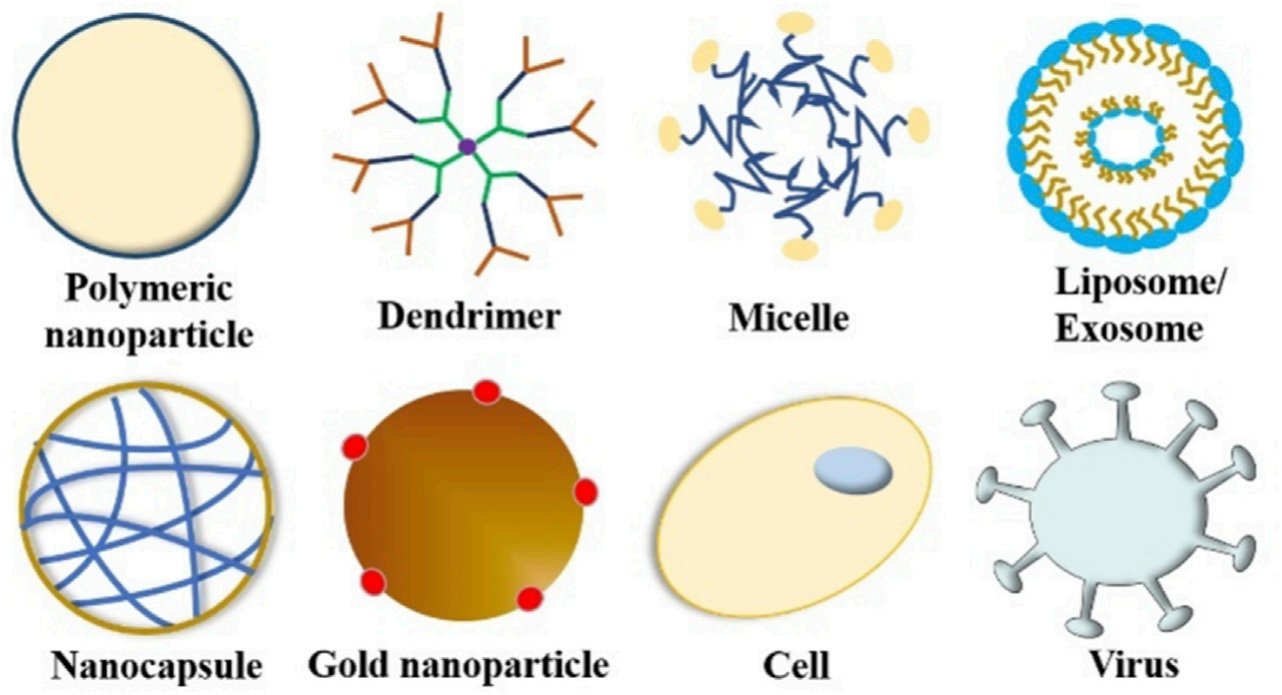
Nanocarriers for drug delivery.

To circumvent these limitations, researchers have developed various strategies and methods to enhance drug delivery efficiency to the CNS, which mainly include bypassing the BBB, crossing the BBB, and BBB manipulation. A few reviews have summarized the nanotherapeutics for GBM treatment based on the classifications of material types, drug targets, and BBB-penetration mechanisms (Jena et al., 2020; Zhao et al., 2020). In this review, based on the classification of therapeutic molecule types, we summarize recent progress in this field from the perspective of tailor-making nanotherapeutics for GBM treatment. An overview of BBB and drug delivery strategies to the CNS (Figure 1), examples of nanocarriers for drug delivery, such as polymeric nanoparticles, liposomes, dendrimer, nanocapsule, and so on (Figure 2), as well as nanotherapeutics that can be delivered across the BBB/BBTB to effectively treat GBM will be provided (Table 1). At last, perspectives on the future development of nanotherapeutics for GBM management will be also provided.

**TABLE 1:** Examples of nanotherapeutics for GBM treatment.

Nanocarrier	Drug	Ligand	Target	Pathway	References
Extracellular vesicle	MTX	L-4F	LDL	RMT	Ye et al. (2018)
Micelle	PTX	TFR-T12	TfR1	RMT	Sun et al. (2020)
Nanoparticle	Panitumumab/TMZ	Panitumumab	EGFR	RMT	Banstola et al. (2020)
Exosomes	DOX	—	—	CMT	Zhang et al. (2021)
Nanoparticle	miR17	FA	FR	RMT	Wang et al. (2013), Wang et al. (2015)
Nanoparticle	siRNA	Ang-2	LRP1	RMT	Zheng et al. (2019)
Nanoparticle	Nimotuzumab	—	NAChRs/ChTs	RMT	Han et al. (2019), Wu et al. (2019a)
Dendrimer	cMBP	—	—	AMT	Wu et al. (2018)
Nanoparticle	—	Ang-2	LRP1	RMT	Tsai et al. (2018)
Gold nanorods	—	RVG	NAChRs	RMT	Lee et al. (2017)
Nanoparticle	—	RGD	α_V_β_3_ IR	RMT	Guo et al. (2018)
Dendrimer	Rapa	—	TAM	CMT	Sharma et al. (2020)
CAR T cell	—	CLTX	Unknown	CMT	Wang et al. (2020)

## Blood-Brain Barrier

BBB consists of brain microvascular endothelial cells (BMECs), which separate the blood in the capillaries from the interstitial fluid (ISF) in the ventricles. There are tight junctions and adhesion junctions formed between adjacent vascular endothelial cells, which effectively block the cracks between BMECs. Under normal circumstances, the effective pore size of BMECs is estimated to be 1.4–1.8 nm, only particles smaller than 1 nm can pass through the pores passively (Sarin, 2010). In addition to the BMECs, the extracellular basement membrane, adjacent pericytes, astrocytes, and microglia are all essential parts of the BBB support system, which form complex and multifunctional “neurovascular unit” (NVU), and also act as a localized immune system (Hawkins and Egleton, 2007; Abbott, 2013). Ions, nutrients, and molecules, which are essential for the regulation of CNS metabolism, are transported by transporters or carriers on the luminal and abluminal membranes for in and out transportation from the brain. At the same time, there are efflux transporters on the lumen and outer membranes, which are mainly from the ATP binding cassette (ABC) transporter family such as P-glycoprotein (P-gp, ABCB1). They can exclude potentially toxic compounds in the circulation, which is also the main reason that potential therapeutic agents failed to penetrate the BBB in history (Qosa et al., 2015). In addition, endothelial cells contain a variety of enzymes, severing as a metabolic barrier to prevent endogenous and exogenous molecules from bypassing the physical barrier imposed by endothelial cells and entering the brain parenchyma to interfere with neuronal function, and inactivate many toxic substances entering the BBB (Zlokovic, 2008). In summary, the function of BBB will seriously affect the efficacy and tolerability of drugs. Understanding the structure and function of BBB can provide theoretical support and ways to overcome BBB and deliver drugs to brain lesions.

Currently, there are three main methods for delivering drugs across BBB to the brain: bypass the blood-brain barrier, cross the blood-brain barrier, or blood-brain barrier manipulation.

### Bypassing the Blood-Brain Barrier

Bypassing the blood-brain barrier includes: 1) Intracerebroventricular (ICV) administration, direct injection of high concentration drugs to lateral ventricle through skull penetration (Bennewitz and Saltzman, 2009); 2) Intraparenchymal administration, direct injection of drugs to brain parenchyma by implantation or injection (Mahoney and Saltzman, 1999); 3) Convection-Enhanced Delivery (CED), in which drug solutions are injected through a small diameter catheter that inserted into the parenchyma, and a positive pressure gradient was applied to the solution by an external pump to make it diffuse (Raghavan et al., 2006); 4) Intrathecal administration, where the therapeutic agent is injected into the subarachnoid space of the spinal cord through a lumbar puncture, and then transported to the CNS substance through CSF (Corning, 1885); All of these methods can bypass the blood-brain barrier and achieve the enrichment of drugs in the brain. However, the high cost of surgical instruments, the invasive nature of these technologies, and the risk of infection have limited their application. Therefore, intratympanic administration and intranasal administration (Balin et al., 1986; Hanson and Frey, 2008; Chen et al., 2010) have been developed as non-invasive alternatives to bypass the BBB and can be combined with nanoparticles for drug delivery, which has broader prospects for the treatment of CNS diseases.

### Crossing the Blood-Brain Barrier

According to the physiological properties of BBB, therapeutic agents can actively cross the BBB into the CNS in four ways (as shown in Figure 1).

1) Carrier-mediated transport (CMT): BMECs express a variety of solute and nutrient transporters, and their inflow and outflow are driven by electrochemical gradient or concentration gradient. Among them, glucose transporter 1 (GLUT1), monocarboxylate transporter 1 (MCT1), large neutral amino acid transporter 1 (LAT1), cationic amino acid transporter type 1 (CAT1), concentrative nucleoside transporter type 2(CNT2) are some of the most famous transporters (Hawkins and Davis, 2005). Efforts can be made to develop chemically modified drugs or nanocarriers to transporting the drug to the brain by CMT and enhancing brain accumulation and selectivity.
2) Receptor-mediated transcytosis (RMT): Large molecules, such as peptides and proteins, pass across the BBB in the form of receptor-mediated endocytosis through various receptors expressed on BMEC. Up to 20 kinds of receptors that can initiate RMT have been identified, some of the well-researched receptors include Transferrin (TfR), LDL receptor-related protein 1 and 2 (LRP-1, LRP-2), Insulin (InsR), Leptin, Epidermal growth factor, and Tumor necrosis factor (Preston et al., 2014). The existence of these carriers and receptors contributes to the application of drug delivery, where the drug can be developed and designed to achieve maximum brain absorption.
3) Adsorptive-mediated transcytosis: The lumen surface of the BMECs is covered by a sugar coating rich in carbohydrates, which makes the lumen surface of BMECs negatively charged (Herve et al., 2008). Therefore, the cationic substance can cross the BBB through electrostatic interaction with the plasma membrane. And for drug design, cations can be introduced or combined with positively charged substances to enter the brain through the AMT pathway. In addition, vesicles formed during the AMT process have a larger capacity than vesicles formed during the RMT process and can hold larger macromolecules.
4) Cell-mediated transport: In inflammatory and disease states, leukocytes directly migrate through the cytoplasm of endothelial cells and penetrate the BBB without destroying tight junctions, and their transport is greatly increased (Bellettato and Scarpa, 2018). Taking advantage of this, drugs or nanoparticles can be designed to stimulate immune cell uptake or simulate activated immune cells to penetrate directly into the diseased area without the need for immune cell uptake (Eniola and Hammer, 2005; Haney et al., 2011).

### Blood-Brain Barrier Manipulation

BBB manipulation mainly involves the opening of tight junctions and the inhibition of efflux pumps. Tight junctions are dynamic complexes of multiple protein components, which can be broken down and reorganized due to various stimuli. External stimuli can be imposed to artificially open tight junctions and facilitate the paracellular delivery of larger compounds (Johnson et al., 2008). External stimuli can be divided into chemical, biological, and physical stimuli, such as hypertonic solutions, ultrasound, and so on. The destruction of BBB must be transient and reversible, otherwise, it may damage the integrity and physiological function of the BBB, leading to the accumulation of potentially harmful substances. In addition, the presence of efflux transporters makes it possible for therapeutic agents to be excreted from the brain even if paracellular transport is increased (Banks, 2016). Therefore, it is important to control the duration of the reversible opening of tight junctions and to ensure that frequent stimulation will not affect the BBB and brain conditions. On the other hand, the presence of efflux proteins on the BBB can prevent many drugs from successfully reaching the brain parenchyma. Anticancer drugs are the first drugs identified as P-gp substrates (Provenzale et al., 2005). To promote treatment, the drug must be chemically modified to prevent it from becoming a P-gp substrate, otherwise P-gp inhibitors must be used. In recent years, it has been demonstrated that the oral bioavailability of efflux pump substrates can be improved by co-administration of efflux pump inhibitors, but it is also necessary to bear the risk of toxicity, accumulation, or off-target (Werle, 2007). The P-gp inhibitor is more suitable for the treatment of acute diseases and can maximize the drug concentration in a relatively short time.

## Nanotherapy for Glioblastoma Treatment

As we mentioned before, the GBM cannot be eliminated by surgery and will recur within a certain period. Radiation therapy may also cause side effects for the powerful beams can potentially damage some surrounding healthy cells. Specifically for patients going through radiation therapy for glioblastoma, they may experience headaches, nausea, vomiting, hearing loss, seizures, and trouble with memory or speech. Under these circumstances, nanotechnologies that enable drug delivery across the BBB hold great promise for the treatment of GBM.

To date, researchers have designed many targeted drug delivery systems with unique targeting and action mechanisms, which can effectively deliver therapeutic drugs across the BBB/BBTB and to the tumor site accurately to achieve a better therapeutic effect. Figure 2 summarizes current nanocarriers commonly used for drug delivery, including polymeric nanoparticles, dendrimer, micelle, liposome/exosome, nanocapsule, gold nanoparticle, cell, and virus. These nanocarriers provide many advantages for drug delivery, such as improved solubility and pharmacokinetics (PK), enhanced efficacy, reduced toxicity, and increased tissue selectivity. In this section, we summarize the nanotechnology-based therapeutic strategies used for GBM treatment, including chemotherapy, gene therapy, protein therapy, phototherapy, and immunotherapy. Examples of nanotherapeutics that can be delivered across the BBB/BBTB to effectively treat GBM are provided in Table 1, wherein the nanocarrier, drug, ligand, target, and pathway used in each nanotherapeutic are detailed.

### Chemotherapy

Small molecule chemotherapeutics, such as doxorubicin (DOX), paclitaxel (PTX), cisplatin (CDDP), erlotinib, methotrexate, etc. (He et al., 2016), are the main drugs used for cancer treatment, but the efficiency is often subjected to systemic toxicity and side effects. With the emerging of nanomedicine, loading chemotherapeutic drugs into nanocarriers for targeted delivery and barriers overcoming has become a promising direction of development.

Transferrin receptor 1 (TfR1) and low-density lipoprotein receptor-related protein-1 (LRP1) are expressed on the BBB, and overexpressed on glioma cells, for which nanocarriers can be designed to target these receptors (Ye et al., 2018; Chan et al., 2021) functionalized the LDLR targeting peptide ApoA-I mimetic peptide (L-4F) to the surface of extracellular vehicles (EVS), which promoted the internalization process mediated by membrane receptors, thereby promoting the transportation of loaded drug methotrexate (MTX) to U87 glioma. Sun et al. (2020) synthesized TFR-T12 peptide-modified polyethylene glycol-polylactic acid (PEG-PLA) micelles as a carrier of paclitaxel (PTX), and the accumulation of TFR-T12 modified micelles in the brain was about 2–3 times higher compared to that of unmodified micelles. However, it is worth noting that the accumulation of TFR-T12 peptide-modified micelles in liver tissue is more than that of unmodified micelles, which might be due to the more presence of TFR in the liver tissue. Banstola et al. (2020) coupled Panitumumab to the surface of polyglycolic acid nanoparticles loaded with temozolomide (PmAb-TMZ-PLGA-NPs), where the panitumumab targets the epidermal growth factor receptor (EGFR), which are overexpressed in glioma. Their approach has been shown to improve the tumor-targeting efficiency while exerting the therapeutic effect of the monoclonal antibody, at the same time increasing the tumor internalization of the nanoparticles and the toxic effect of temozolomide. PmAb-TMZ-PLGA-NPs have a more obvious cytotoxic effect in the GBM model (U-87 MG) with high EGFR expression than the low EGFR GBM model (LN229). Exosomes are extracellular microcapsules driven from cells, which can be severed as drug carriers to cross biological barriers in the body. Zhang et al. (2021) loaded DOX into exosomes isolated from bEnd cells. Their *in-vitro* transwell experiment showed that the penetration rate was about three times higher than that of DOX alone.

In addition, to further improve the efficiency of brain penetration, researchers have combined receptor targeting peptides with cell-penetrating peptides (Zhu et al., 2018; Lakkadwala et al., 2019). Cell-penetrating peptides (CPPs) are short-chain cationic peptides that can be transported across biological membranes and carry cargoes within living cells without destroying the integrity of cell membranes. The maximum tumor accumulation can reach ∼5.79% ID/gram of tissue, which is more than 12 times higher than that of chemotherapy alone (Lakkadwala et al., 2019).

### Gene Therapy

Gene therapy is considered to be one of the most promising methods for the treatment of malignant tumors (Liu et al., 2015; Zhang et al., 2015; Meng et al., 2016a; Liu et al., 2019b; Liu et al., 2021a). miRNAs include a new class of small non-coding endogenous RNAs that regulate gene expression by directing their target mRNA degradation or translational inhibition. An attractive feature of using miRNAs as therapeutic agents is that they can target multiple genes and effectively regulate different biological processes in the environment. Small interfering RNA (siRNA) is a non-coding double-stranded RNA molecule that is similar to miRNA, with a short and well-defined structure of usually around 20–24 base pairs. Due to its high specificity, low dose requirements, and relatively simple drug development process, it has inherent advantages and great potential in the treatment of refractory diseases. CRISPR (clustered regularly interspaced short palindromic repeats) is a family of DNA sequences found in the genomes of prokaryotic organisms such as bacteria and archaea. In the presence of Cas9 (a bacterially derived enzyme that snips DNA) and a synthetic guide RNA, CRISPR work as a RNA-guided gene-editing platform to introduce a double-strand break at a specific location within the genome. The delivery of CRISPR/Cas9 and genes is also a novel antitumor strategy to overcome tumor heterogeneity (Liu et al., 2019a; Liu et al., 2021b).

However, there is currently a lack of suitable RNA delivery systems to show good circulatory stability and effective targeted delivery ability, which hinders the therapeutic effect of RNA. Zhang group (Wang et al., 2013; Wang et al., 2015) extracted lipids from the tissues of grapefruit and reassembled them into nanoparticles. They were called grapefruit-derived nanocarriers (GNV), which have a multi-layered flower-like structure that can efficiently deliver a variety of therapeutic agents without causing toxicity, including chemotherapeutics, siRNA, and antibodies. In a recent study, GNV was coated with folic acid to achieve precise targeting of folate receptor-positive GL-26 brain tumors (Zhuang et al., 2016). Since high-affinity folate receptors (FRs) have increased expression levels on many human tumors, the expression levels on non-tumor cells are almost negligible. FA-GNV is loaded with miR17, which displays a therapeutic effect mainly through inducing the production of NK cells by down-regulating the expression of MHC1 on the surface of GL-26 tumor cells. This drug was administered by nasal injection to mice. Twelve hours after nasal injection, DIR labeled GNVs were observed in the brain, mainly in the olfactory bulb, hippocampus, thalamus, and cerebellum, while little fluorescence was detected in the brains of mice injected with PBS or free DIR dye. FA-pGNV/miR17 treatment can increase the number of DX5+NK cells in GL-26 tumors in mice. However, miR17, like other miRNAs, is a pleiotropic miRNA that can target multiple pathways, the contribution of other mechanisms against tumor growth cannot be ruled out.

Recent reports have shown that polymers with guanidine (Gu) groups can easily adhere to siRNA by forming Gu^+^/PO_3_^4−^ salt bridges. Shi group (Zheng et al., 2019) introduced the Gu^+^/PO_3_^4−^ salt bridge and hydrophobic interaction into the formulation of polymer nanomedicine at the same time, and the resulting “triple interaction” siRNA nanomedicine showed excellent stability. Nanoparticles are formed by self-assembling of poly(ethylene glycol)-block-poly[(N-(3-methacrylamidopropyl) guanidinium-co-4-(4,4,5,5-tetramthyl-1,3,2-dioxaborolan-2-yl)benzyl acrylate )] (PEG-bP(Gu/Hb))/Ang-poly(ethylene glycol)-block-poly(N-(3-methacryl-amidopropyl) guanidinium) (Ang-PEG-b-PGu) with siRNA, and the introduction of angiopep-2 ligand increased BBB penetration and GBM targeting. When nanoparticles are enriched in the tumor microenvironment with a high ROS level, the hydrophobic phenylboronic acid ester is converted into a hydrophilic counterpart with carboxyl groups, which interferes with electrostatic and hydrogen bond interactions so that siRNA can be effectively released. In an *in-situ* mice U87 tumor model, active targeting and combined RNAi treatment significantly prolonged the survival time of mice, with a median survival time of 36 days (18 days in the control group). In addition, they also designed a multifunctional nanocapsule containing a single siRNA, which achieved the preparation of small-sized siRNA nanocapsules (∼25.3 nm) and nearly 100% siRNA encapsulation efficiency (Zou et al., 2020).

### Protein Therapy

Protein, especially therapeutic antibodies, have shown advantages in controlling primary tumors. Since they cannot cross the BBB, the concentration of monoclonal antibodies that can be delivered to the brain is usually 1,000 times lower than their concentration in the blood (Shah and Betts, 2013). Such a low concentration in the brain cannot meet the required therapeutic level in the tumor lesions and is ineffective for the treatment of brain tumors. In addition, due to their large size in molecules, it is difficult to design a nanocarrier system with high payload capacity without damaging the biological activity.

Nicotinic acetylcholine transporters (NAChRs) and choline transporters (ChTs) are widely expressed in the nervous system and brain capillary endothelial cells, choline, and acetylcholine are actively transported to the brain from the blood circulation. Inspired by the active transport of choline and acetylcholine, Lu group (Han et al., 2019; Wen et al., 2019; Wu et al., 2019a; Xu et al., 2019a; Qin et al., 2020) designed a 2-methacryloyloxyethyl phosphorylcholine (MPC) based polymer system called nanocapsules. MPC is an analog of choline and acetylcholine, which can interact with NAChRs and ChTs in a manner similar to acetylcholine and choline, and can maintain a long-term circulation in the blood by resisting protein adsorption and macrophage phagocytosis (Liu et al., 2012; Long et al., 2016; Li et al., 2019; Wu et al., 2019b). Using MPC as a monomer and combining it with a degradable or non-degradable crosslinking agent, a thin polymer network is grown around a single protein molecule through *in-situ* free radical polymerization. Under the mediation of nAChRs and ChTs, the nanocapsules can be effectively transported through the blood-brain barrier to reach the brain. Bovine serum albumin (BSA) was used as a model protein to verify its ability to penetrate BBB. The fluorescence images of the main organs of mice after intravenous injection showed that the accumulation of BSA nanocapsules (denoted as n(BSA)) in the brain was 42 times higher than that of native BSA. Immunofluorescence histopathological analysis showed that the nanocapsules effectively penetrated the BBB and were evenly distributed in the extracellular space of neurons 1 day after i.v. of n(BSA). Nimotuzumab (Nimo) targets epidermal growth factor receptor (EGFR) and is in the late stage of a clinical trial of high-grade glioma (Bode et al., 2012). Nimo was selected as a therapeutic agent and combined with the characteristics of high expression of matrix metalloproteinase-2 (MMP-2) in the brain tumor microenvironment. n(Nimo) was synthesized by the polymerization of MPC and MMP-2 responsive peptide crosslinker. Twelve hours after intravenous injection, the concentration of nimotuzumab in mice CSF was 0.084 µg/ml, equivalent to 0.1% of plasma concentration, while n(Nimo) group (0.85 µg/ml) was 10 times higher than that of native nimotuzumab group, which was equivalent to 1.1% of plasma concentration. In order to verify that nanocapsules enter the brain through choline transporter, mice were intraperitoneally injected with gradient choline transporter inhibitor, hemicholinium-3 (HC-3), before administration. The increased dose of HC-3 significantly reduced the fluorescence intensity in the brain of glioma-bearing mice. In orthotopic mice U87-EGFRwt glioma xenograft, the expression level of p-EGFR (activated form of EGFR) in tumor tissues treated with n(Nimo) was significantly lower, which was about 40% of that in mice treated with native nimotuzumab. The percentage of Ki67-positive cells (the main marker of tumor proliferation) also decreased from ∼80 to ∼30%, confirming that n(Nimo) has excellent antitumor effects.

Besides monoclonal antibodies, peptides have been widely used in the prevention, diagnosis, and treatment of tumors and other diseases, for their lower price, easy to mass production, low immunogenicity, and stronger permeability in tissues (Habra et al., 2021). The human mesenchymal-epithelial conversion factor (MET) proto-oncogene is located on chromosome 7q31. More and more researches have shown that MET plays a critical role in the proliferation, survival, migration, invasion, angiogenesis, stem cell characteristics, therapeutic resistance, and recurrence of glioblastomas. The expression of pMET in GBM patients was 28.7 times more than that of the corresponding normal brain tissues (Cheng and Guo, 2019). Wu et al. (2018) coupled the cMBP peptide, a MET target peptide, to the surface of the PAMAM-NH_2_ G4 dendrimer as a new type of nanoinhibitor. And the positive charges on the nanoinhibitor promote their penetration through BBB by AMT pathway. Compared with the binding affinity of the free peptide (Kd = 3.96 × 10^−7^ M), the binding affinity of the nanoinhibitor to MET increased by three orders of magnitude, reaching 1.32 × 10^−10^ M. In mice U87 MG xenograft tumor model, pMET levels decreased by 71.0% at 2 h after intravenous injection of nanoinhibitors, while pMET levels decreased by less than 22.0% after taking free cMBP polypeptide. The results showed that the nanoinhibitor effectively targeted the brain and inhibited the expression of pMET.

### Phototherapy

Phototherapy including photodynamic therapy (PDT) and photothermal therapy (PTT), has attracted great interest due to its low invasiveness and fewer side effects compared with traditional cancer treatment methods. PDT uses photosensitizer (PS) to irradiate the tumor cells in aerobic conditions to produce reactive oxygen species (ROS), which can induce apoptosis or necrosis of tumor cells. PTT is based on materials with high photothermal conversion efficiency, which converts energy from light (usually near-infrared) into heat to kill cancer cells (Felsher, 2003; Wu et al., 2020). In 1993, the regulatory authority approved PDT, namely Photofrin (PF), for the first time to treat bladder cancer in specific cases (Celli et al., 2010). However, the effect of PDT is limited by the hypoxic environment in the tumor. Even though PTT does not depend on oxygen, the excessive temperature (>50°C) during PTT may cause inevitable damage to the surrounding normal tissues. Phototherapy has not been accepted as a first-line tumor intervention.

The application of nanoparticles in phototherapy is a big step forward in solving some of these related challenges. Tsai et al. (2018) developed an angiopep-2 coupled upconversion nanoparticle, Ang-IMNPs, which simultaneously carried photothermal/photodynamic sensitizer (IR-780/5,10,15,20-tetrakis(3-hydroxyphenyl) chlorin (mTHPC)), for the synergistic treatment of GBM. In an *in situ* ALTS1C1 mice brain tumor model, the accumulation of Ang-IMNPs increased significantly at 8 hours after intravenous injection, which was about 2.3 times higher than that of IMNPs. Due to the inherent invasiveness of ALTS1C1 astrocytoma cells in brain tumors, the median survival time is only 8 days in the PBS group, and only 14 days after Ang-IMNP treatment without radiation. After receiving 980/808 nm laser combined irradiation the median survival time extended to 24 days.

Rabies virus is a typical neurotropic virus, and the rabies virus glycoprotein (RVG) enables the virus to enter the CNS in an RMT pathway by binding to the nicotinic acetylcholine receptor (NAChRs). Inspired by it, Lee et al. (2017) simulated the structure and function of the rabies virus and designed silicone coated gold nanorods (RVG-PEG-AuNRs@SiO_2_). Generally, gold-based nanomaterials (GBNs) have the advantages of good biocompatibility, low immunogenicity, high physiological stability, and controllable size and surface properties (Tu et al., 2021). Four hours after i.v. injection to N2a tumor-bearing mice, 808 nm laser was used to irradiate the tumor site, RVG-PEG-AuNRs@SiO_2_ significantly inhibited the growth of mouse tumors. 7 days after treatment, the tumor volume of the RVG-PEG-AuNRs@SiO_2_ group was significantly smaller than that of the PEG-AuNRs@SiO_2_ group and the saline control group (124.8 ± 147.5, 1,067.4 ± 295.4, and 2,323.2 ± 436.3 mm^3^, respectively), which showed excellent brain tumor treatment potential. The α_V_β_3_ integrin receptor is also overexpressed in brain tumor vascular endothelial cells and glioblastoma cells. Guo et al. (2018) prepared nanoparticle P1 by copolymerizing electron-rich donor unit alkyl-chain-grafted BDT and electron-deficient receptor unit BBT, and the α_V_β_3_ integrin receptor target RGD peptide was coupled to the surface of the nanoparticle (P1-NP). Under laser irradiation (1,064 nm, 1 W/cm^2^), the temperature of P1-NP rises rapidly, reaching a plateau of 64.8°C at t = 5 min, and has a photothermal conversion efficiency of 30.1% which showed a great photothermal conversion ability. After P1-RGD-NP plus laser treatment, tumor temperature rose rapidly from 36.8 to 52.8°C, while adjacent normal tissues only rose to 40.5°C with little change. H&E staining showed serious tumor tissue damages and a high cell necrosis rate.

### Immunotherapy

The interaction between tumor cells and the immune system is the main determinant of cancer. Immunotherapy, as one of the fastest-growing cancer treatment options, has demonstrated its efficacy on various types of cancers (Sharma et al., 2019). Immune checkpoint inhibitors such as PD-1, PD-L1, and CTLA-4 promote the normal balance of the adaptive immune system to enhance immune activation, FDA has approved the marketing of ipilimumab, pembrolizumab, nivolumab, etc (Preusser et al., 2015). However, malignant glioma is one of the most severely immunosuppressed solid tumors (Pakawat et al., 2018). Currently, there is no FDA-approved immunotherapy for GBM, and several GBM phase 3 immunotherapy clinical trials have failed. The most promising strategy for GBM immunotherapy deems to be a combination of immunotherapy with other types of treatment to overcome the severe immunosuppression of this disease. For example, the combination of chemotherapy [doxorubicin (Kinoh et al., 2020), carmustine (Mathios et al., 2016)], and anti- PD1 therapy can trigger immunogenic cell death (ICD) and enhance immune response. A combination of VEGF and Ang-2 can effectively target the blood vessels and immune cells in the GBM model and reduce immunosuppression (Di Tacchio et al., 2019). Combination with other immune checkpoint inhibitors (Hung et al., 2018) can overcome the up-regulation of other inhibitory checkpoints blocked by a single checkpoint and drug resistance.

Galstyan et al. (2019) covalently linked CTLA-4 and PD-1 antibodies to the poly (β-L-malic acid) (PMLA) backbone (named NICs). NICs can cross the BBB through transferrin receptor (TfR)-mediated transcytosis, thereby delivering antibodies to tumor sites, activating local immune responses, and achieving the purpose of treating brain tumors. The NIC combination therapy has obvious advantages in recruiting CD8^+^ T cells into tumor tissues, enhancing cytotoxic immune response and systemic immune response. Xu et al. (2019b) found that the photosensitizer chlorin e6 (Ce6) and immunoglobulin G (IgG) inherently bind within the nano-level affinity range. Ce6 and IgG spontaneously assemble in the presence of the pharmaceutical excipient polyvinylpyrrolidone (PVP) and form a nanostructure (about 30 nm), called Chloringlobulin (Chlorin e6 + immunoglobulin G). In their research, the immune checkpoint inhibitor anti-programmed death-ligand 1 (PD-L1) (αPD-L1) was used to prepare αPD-L1 Chloringlobulin, which can combine photodynamic therapy and PD-L1 blockade therapy to the treatment of glioma. After the combination therapy, the infiltration of CD8^+^ T cells and NK cells in the tumor was enhanced most significantly, the median survival time was prolonged to 32 days (23 days in the PDT single-agent group, 27 days in the αPD-L1 single-agent group), resulting in a long-term memory response. Nanomaterials can improve the efficacy and reduce potential off-target and side effects in immunotherapy, but further efforts are needed to include them in the next generation of immunotherapy. In addition, there is no standardized and effective method to measure immune response, the management of immune-related adverse events in the CNS is still a problem.

In addition, therapy based on cells (Sharma et al., 2020; Wang et al., 2020) (CAR T cell, tumor-associated macrophage) and oncolytic virus 2018; Yoo et al. (2019) provides an innovative idea for the treatment of GBM and bridging the gap between clinical needs and effective treatment. The combination of chemotherapy, radiotherapy, gene therapy, and monoclonal antibody, and so on (Qian et al., 2013; Shi et al., 2013; Qian et al., 2014; Ren et al., 2014; Meng et al., 2016a; Meng et al., 2016b; Qi et al., 2016; Ren et al., 2016; Ashton et al., 2018; Desjardins et al., 2018; Jia et al., 2018; Zhao et al., 2018; Liu et al., 2019a; Qian et al., 2019; Zhan et al., 2020; Qi et al., 2021)can reduce the systemic dosage and related side effects, broaden the treatment window, and rejuvenate some candidate drugs that are on the verge of failure. Certainly, combination therapy is not just a simple combination of several methods, a lot of preclinical researches must be performed to find suitable drugs, medication time points and the treatment sequence. Nonetheless, the combination therapy enriches the human “arms” of anti-cancer, provides doctors with more choices, and brings more hope to patients.

## Conclusion

Despite that much progress has been made, there is no treatment better than the standard treatment for GBM represented by temozolomide/radiotherapy which has an average survival time of 14.6 months (Mcgranahan et al., 2019) to date. New therapies should effectively overcome the blockage of the BBB, release the loaded therapeutic drugs, and inhibit tumor cells, thus improving the survival and life quality of patients. As a new kind of materials, nanomaterials play an important role in the delivery of various drugs, with the ability of transmission through biological barriers and precise tumor targeting, which provides more opportunities for the creation of advanced therapies for brain tumor treatment. But there are still limitations to the use of nanomaterials, such as the poor stability of liposomes, poor biocompatibility and circulation of inorganic nanoparticles, and biosecurity of viruses and cells. More efforts are still needed to clarify the toxicology, stability, safety, and clearance mechanism to shift the clinical translation of nanotherapeutics for glioma treatment (Chen et al., 2018).

## References

[B1] Abbott N. J. (2013). Blood-Brain Barrier Structure and Function and the Challenges for CNS Drug Delivery. J. Inherit. Metab. Dis. 36, 437–449. 10.1007/s10545-013-9608-0 23609350

[B2] Anjum K. Shagufta B. I. Abbas S. Q. Patel S. Khan I. Shah S. A. A. (2017). Current Status and Future Therapeutic Perspectives of Glioblastoma Multiforme (GBM) Therapy: A Review. Biomed. Pharmacother. 92, 681–689. 10.1016/j.biopha.2017.05.125 28582760

[B3] Ashton J. R. Castle K. D. Qi Y. Kirsch D. G. West J. L. Badea C. T. (2018). Dual-Energy CT Imaging of Tumor Liposome Delivery After Gold Nanoparticle-Augmented Radiation Therapy. Theranostics 8, 1782–1797. 10.7150/thno.22621 29556356 PMC5858500

[B4] Balin B. J. Broadwell R. D. Salcman M. el-Kalliny M. (1986). Avenues for Entry of Peripherally Administered Protein to the central Nervous System in Mouse, Rat, and Squirrel Monkey. J. Comp. Neurol. 251, 260–280. 10.1002/cne.902510209 3782501

[B5] Banks W. A. (2016). From Blood-Brain Barrier to Blood-Brain Interface: New Opportunities for CNS Drug Delivery. Nat. Rev. Drug Discov. 15, 275–292. 10.1038/nrd.2015.21 26794270

[B6] Banstola A. Duwa R. Emami F. Jeong J. H. Yook S. (2020). Enhanced Caspase-Mediated Abrogation of Autophagy by Temozolomide-Loaded and Panitumumab-Conjugated Poly(lactic-co-Glycolic Acid) Nanoparticles in Epidermal Growth Factor Receptor Overexpressing Glioblastoma Cells. Mol. Pharm. 17, 4386–4400. 10.1021/acs.molpharmaceut.0c00856 33079558

[B7] Bellettato C. M. Scarpa M. (2018). Possible Strategies to Cross the Blood-Brain Barrier. Ital. J. Pediatr. 44, 131. 10.1186/s13052-018-0563-0 30442184 PMC6238258

[B8] Bennewitz M. F. Saltzman W. M. (2009). Nanotechnology for Delivery of Drugs to the Brain for Epilepsy. Neurotherapeutics 6, 323–336. 10.1016/j.nurt.2009.01.018 19332327 PMC2673491

[B9] Bode U. Massimino M. Bach F. Zimmermann M. Khuhlaeva E. Westphal M. (2012). Nimotuzumab Treatment of Malignant Gliomas. Expert Opin. Biol. Ther. 12, 1649–1659. 10.1517/14712598.2012.733367 23043252

[B10] Celli J. P. Spring B. Q. Rizvi I. Evans C. L. Samkoe K. S. Verma S. (2010). Imaging and Photodynamic Therapy: Mechanisms, Monitoring, and Optimization. Chem. Rev. 110, 2795–2838. 10.1021/cr900300p 20353192 PMC2896821

[B11] Chan M. H. Chen W. Li C. H. Fang C. Y. Chang Y. C. Wei D. H. (2021). An Advanced *In Situ* Magnetic Resonance Imaging and Ultrasonic Theranostics Nanocomposite Platform: Crossing the Blood-Brain Barrier and Improving the Suppression of Glioblastoma Using Iron-Platinum Nanoparticles in Nanobubbles. ACS Appl. Mater. Inter. 13, 26759–26769. 10.1021/acsami.1c04990 34076419

[B12] Chen G. Zhang X. Yang F. Mu L. (2010). Disposition of Nanoparticle-Based Delivery System via Inner Ear Administration. Curr. Drug Metab. 11, 886–897. 10.2174/138920010794479673 21208174

[B13] Chen Q. Wu J. Ye Q. Ma F. Zhu Q. Wu Y. (2018). Treatment of Human Glioblastoma with a Live Attenuated Zika Virus Vaccine Candidate. mBio 9 (5), e01683–18. 10.1128/mBio.01683-18 30228241 PMC6143740

[B14] Cheng F. Guo D. (2019). MET in Glioma: Signaling Pathways and Targeted Therapies. J. Exp. Clin. Cancer Res. 38, 270. 10.1186/s13046-019-1269-x 31221203 PMC6585013

[B15] Corning J. L. (1885). Spinal Anaesthesia and Local Medication of the Cord. N Y Med. J. 42, 483–485.

[B16] Delgado-López P. D. Corrales-García E. M. (2016). Survival in Glioblastoma: a Review on the Impact of Treatment Modalities. Clin. Transl. Oncol. 18, 1062–1071. 10.1007/s12094-016-1497-x 26960561

[B17] Desjardins A. Gromeier M. Herndon J. E. 2nd Beaubier N. Bolognesi D. P. Friedman A. H. (2018). Recurrent Glioblastoma Treated With Recombinant Poliovirus. N. Engl. J. Med. 379, 150–161. 10.1056/NEJMoa1716435 29943666 PMC6065102

[B18] Di Tacchio M. Macas J. Weissenberger J. Sommer K. Bähr O. Steinbach J. P. (2019). Tumor Vessel Normalization, Immunostimulatory Reprogramming, and Improved Survival in Glioblastoma with Combined Inhibition of PD-1, Angiopoietin-2, and VEGF. Cancer Immunol. Res. 7, 1910–1927. 10.1158/2326-6066.CIR-18-0865 31597643

[B19] Eniola A. O. Hammer D. A. (2005). Characterization of Biodegradable Drug Delivery Vehicles with the Adhesive Properties of Leukocytes II: Effect of Degradation on Targeting Activity. Biomaterials 26, 661–670. 10.1016/j.biomaterials.2004.03.003 15282144

[B20] Esteller M. Garcia-Foncillas J. Andion E. Goodman S. N. Hidalgo O. F. Vanaclocha V. (2000). Inactivation of the DNA-Repair Gene MGMT and the Clinical Response of Gliomas to Alkylating Agents. N. Engl. J. Med. 343, 1350–1354. 10.1056/NEJM200011093431901 11070098

[B21] Felsher D. W. (2003). Cancer Revoked: Oncogenes as Therapeutic Targets. Nat. Rev. Cancer 3, 375–380. 10.1038/nrc1070 12724735

[B22] Galstyan A. Markman J. L. Shatalova E. S. Chiechi A. Korman A. J. Patil R. (2019). Blood-Brain Barrier Permeable Nano Immunoconjugates Induce Local Immune Responses for Glioma Therapy. Nat. Commun. 10, 3850. 10.1038/s41467-019-11719-3 31462642 PMC6713723

[B23] Guo B. Sheng Z. Hu D. Liu C. Zheng H. Liu B. (2018). Through Scalp and Skull NIR-II Photothermal Therapy of Deep Orthotopic Brain Tumors with Precise Photoacoustic Imaging Guidance. Adv. Mater. 30, e1802591. 10.1002/adma.201802591 30129690

[B24] Habra K. Mcardle S. E. B. Morris R. H. Cave G. W. V. (2021). Synthesis and Functionalisation of Superparamagnetic Nano-Rods towards the Treatment of Glioblastoma Brain Tumours. Nanomaterials 11, 2157. 10.3390/nano11092157 34578472 PMC8472662

[B25] Han L. Liu C. Qi H. Zhou J. Wen J. Wu D. (2019). Systemic Delivery of Monoclonal Antibodies to the Central Nervous System for Brain Tumor Therapy. Adv. Mater. 31, e1805697. 10.1002/adma.201805697 30773720

[B26] Haney M. J. Zhao Y. Li S. Higginbotham S. M. Booth S. L. Han H. Y. (2011). Cell-Mediated Transfer of Catalase Nanoparticles from Macrophages to Brain Endothelial, Glial and Neuronal Cells. Nanomedicine 6, 1215–1230. 10.2217/nnm.11.32 21449849 PMC3166447

[B27] Hanson L. R. Frey W. H. 2nd (2008). Intranasal Delivery Bypasses the Blood-Brain Barrier to Target Therapeutic Agents to the central Nervous System and Treat Neurodegenerative Disease. BMC Neurosci. 9 (3), S5. 10.1186/1471-2202-9-S3-S5 PMC2604883 19091002

[B28] Hawkins B. T. Davis T. P. (2005). The Blood-Brain Barrier/Neurovascular Unit in Health and Disease. Pharmacol. Rev. 57, 173–185. 10.1124/pr.57.2.4 15914466

[B29] Hawkins B. T. Egleton R. D. (2007). Pathophysiology of the Blood-Brain Barrier: Animal Models and Methods. Curr. Top. Dev. Biol. 80, 277–309. 10.1016/S0070-2153(07)80007-X 17950377

[B30] He C. Tang Z. Tian H. Chen X. (2016). Co-Delivery of Chemotherapeutics and Proteins for Synergistic Therapy. Adv. Drug Deliv. Rev. 98, 64–76. 10.1016/j.addr.2015.10.021 26546464

[B31] Hegi M. E. Diserens A. C. Godard S. Dietrich P. Y. Regli L. Ostermann S. (2004). Clinical Trial Substantiates the Predictive Value of O-6-Methylguanine-DNA Methyltransferase Promoter Methylation in Glioblastoma Patients Treated with Temozolomide. Clin. Cancer Res. 10, 1871–1874. 10.1158/1078-0432.ccr-03-0384 15041700

[B32] Hervé F. Ghinea N. Scherrmann J. M. (2008). CNS Delivery via Adsorptive Transcytosis. AAPS J. 10, 455–472. 10.1208/s12248-008-9055-2 18726697 PMC2761699

[B33] Hung A. L. Maxwell R. Theodros D. Belcaid Z. Mathios D. Luksik A. S. (2018). TIGIT and PD-1 Dual Checkpoint Blockade Enhances Antitumor Immunity and Survival in GBM. Oncoimmunology 7, e1466769. 10.1080/2162402X.2018.1466769 30221069 PMC6136875

[B34] Jena L. Mcerlean E. Mccarthy H. (2020). Delivery across the Blood-Brain Barrier: Nanomedicine for Glioblastoma Multiforme. Drug Deliv. Transl Res. 10, 304–318. 10.1007/s13346-019-00679-2 31728942 PMC7066289

[B35] Jia G. Han Y. An Y. Ding Y. He C. Wang X. (2018). NRP-1 Targeted and Cargo-Loaded Exosomes Facilitate Simultaneous Imaging and Therapy of Glioma *In Vitro* and *In Vivo* . Biomaterials 178, 302–316. 10.1016/j.biomaterials.2018.06.029 29982104

[B36] Johnson P. H. Frank D. Costantino H. R. (2008). Discovery of Tight junction Modulators: Significance for Drug Development and Delivery. Drug Discov. Today 13, 261–267. 10.1016/j.drudis.2007.10.023 18342803

[B37] Kinoh H. Quader S. Shibasaki H. Liu X. Maity A. Yamasoba T. (2020). Translational Nanomedicine Boosts Anti-PD1 Therapy to Eradicate Orthotopic PTEN-Negative Glioblastoma. ACS Nano 14, 10127–10140. 10.1021/acsnano.0c03386 32806051

[B38] Lakkadwala S. Dos Santos Rodrigues B. Sun C. Singh J. (2019). Dual Functionalized Liposomes for Efficient Co-Delivery of Anti-Cancer Chemotherapeutics for the Treatment of Glioblastoma. J. Control. Release 307, 247–260. 10.1016/j.jconrel.2019.06.033 31252036 PMC6732022

[B39] Lee C. Hwang H. S. Lee S. Kim B. Kim J. O. Oh K. T. (2017). Rabies Virus-Inspired Silica-Coated Gold Nanorods as a Photothermal Therapeutic Platform for Treating Brain Tumors. Adv. Mater. 29 (13), 1605563. 10.1002/adma.201605563 28134459

[B40] Li S. Chen L. Huang K. Chen N. Zhan Q. Yi K. (2019). Tumor Microenvironment‐Tailored Weakly Cell‐Interacted Extracellular Delivery Platform Enables Precise Antibody Release and Function. Adv. Funct. Mater. 29 (43), 1903296. 10.1002/adfm.201903296

[B41] Liu C. Long L. Li Z. He B. Wang L. Wang J. (2012). Hollow poly(MPC-g-PEG-b-PLA) Graft Copolymer Microcapsule as a Potential Drug Carrier. J. Microencapsul 29, 242–249. 10.3109/02652048.2011.646328 22214322

[B42] Liu C. Wen J. Meng Y. Zhang K. Zhu J. Ren Y. (2015). Efficient Delivery of Therapeutic miRNA Nanocapsules for Tumor Suppression. Adv. Mater. 27, 292–297. 10.1002/adma.201403387 25400269

[B43] Liu C. Wen J. Li D. Qi H. Nih L. Zhu J. (2021a). Systemic Delivery of microRNA for Treatment of Brain Ischemia. Nano Res. 14, 3319–3328. 10.1007/s12274-021-3413-8

[B44] Liu Q. Cai J. Zheng Y. Tan Y. Wang Y. Zhang Z. (2019a). NanoRNP Overcomes Tumor Heterogeneity in Cancer Treatment. Nano Lett. 19, 7662–7672. 10.1021/acs.nanolett.9b02501 31593471

[B45] Liu Q. Zhao K. Wang C. Zhang Z. Zheng C. Zhao Y. (2019b). Multistage Delivery Nanoparticle Facilitates Efficient CRISPR/dCas9 Activation and Tumor Growth Suppression *In Vivo* . Adv. Sci. 6, 1801423. 10.1002/advs.201801423 PMC6325604 30643726

[B46] Liu Q. Zhang T. Zheng Y. Wang C. Shi L. (2021b). Calixarene-Embedded Nanoparticles for Interference-free Gene–Drug Combination Cancer Therapy. Small 17 (8), e2006223. 10.1002/smll.202006223 33522123

[B47] Long L.-X. Zhao J. Li K. He L.-G. Qian X.-M. Liu C.-Y. (2016). Synthesis of Star-Branched PLA-B-PMPC Copolymer Micelles as Long Blood Circulation Vectors to Enhance Tumor-Targeted Delivery of Hydrophobic Drugs *In Vivo* . Mater. Chem. Phys. 180, 184–194. 10.1016/j.matchemphys.2016.05.062

[B48] Mahoney M. J. Saltzman W. M. (1999). Millimeter-Scale Positioning of a Nerve-Growth-Factor Source and Biological Activity in the Brain. Proc. Natl. Acad. Sci. U.S.A. 96, 4536–4539. 10.1073/pnas.96.8.4536 10200297 PMC16367

[B49] Mathios D. Kim J. E. Mangraviti A. Phallen J. Park C. K. Jackson C. M. (2016). Anti-PD-1 Antitumor Immunity is Enhanced by Local and Abrogated by Systemic Chemotherapy in GBM. Sci. Transl Med. 8, 370ra180. 10.1126/scitranslmed.aag2942 PMC5724383 28003545

[B50] Mcgranahan T. Therkelsen K. E. Ahmad S. Nagpal S. (2019). Current State of Immunotherapy for Treatment of Glioblastoma. Curr. Treat. Options. Oncol. 20, 24. 10.1007/s11864-019-0619-4 30790064 PMC6394457

[B51] Meng Y. Li X. Li Z. Liu C. Zhao J. Wang J. (2016a). Surface Functionalization of Titanium Alloy with miR-29b Nanocapsules to Enhance Bone Regeneration. ACS Appl. Mater. Inter. 8, 5783–5793. 10.1021/acsami.5b10650 26887789

[B52] Meng Y. Liu C. Zhao J. Li X. Li Z. Wang J. (2016b). An Injectable miRNA-Activated Matrix for Effective Bone Regeneration *In Vivo* . J. Mater. Chem. B 4, 6942–6954. 10.1039/c6tb01790h 32263561

[B53] Miller K. Wang M. Gralow J. Dickler M. Cobleigh M. Shenkier T. (2008). Paclitaxel Plus Bevacizumab Versus Paclitaxel Alone for Metastatic Breast Cancer. J. Evid. Based Med. 19, 272–273. 10.1056/NEJMoa072113 18160686

[B54] Ningaraj N. S. Rao M. Hashizume K. Asotra K. Black K. L. (2002). Regulation of Blood-Brain Tumor Barrier Permeability by Calcium-Activated Potassium Channels. J. Pharmacol. Exp. Ther. 301, 838–851. 10.1124/jpet.301.3.838 12023511

[B55] Ostrom Q. T. Patil N. Cioffi G. Waite K. Kruchko C. Barnholtz-Sloan J. S. (2020). CBTRUS Statistical Report: Primary Brain and Other Central Nervous System Tumors Diagnosed in the United States in 2013-2017. Neuro Oncol. 22, iv1–iv96. 10.1093/neuonc/noaa200 33123732 PMC7596247

[B56] Pakawat C. Christina J. Shohei K. Franziska L. Cui X. Harrison F. S. (2018). Sequestration of T Cells in Bone Marrow in the Setting of Glioblastoma and Other Intracranial Tumors. Nat. Med. 24 (9), 1459–1468. 10.1038/s41591-018-0135-2 30104766 PMC6129206

[B57] Preston J. E. Joan Abbott N. Begley D. J. (2014). “Transcytosis of Macromolecules at the Blood-Brain Barrier,” in Pharmacology of the Blood Brain Barrier: Targeting CNS Disorders (Cambridge, MA: Academic Press), 147–163. 10.1016/bs.apha.2014.06.001 25307216

[B58] Preusser M. Lim M. Hafler D. A. Reardon D. A. Sampson J. H. (2015). Prospects of Immune Checkpoint Modulators in the Treatment of Glioblastoma. Nat. Rev. Neurol. 11, 504–514. 10.1038/nrneurol.2015.139 26260659 PMC4782584

[B59] Provenzale J. M. Mukundan S. Dewhirst M. (2005). The Role of Blood-Brain Barrier Permeability in Brain Tumor Imaging and Therapeutics. AJR Am. J. Roentgenol. 185, 763–767. 10.2214/ajr.185.3.01850763 16120931

[B60] Qi H. Liu C. Long L. Ren Y. Zhang S. Chang X. (2016). Blood Exosomes Endowed With Magnetic and Targeting Properties for Cancer Therapy. ACS Nano 10, 3323–3333. 10.1021/acsnano.5b06939 26938862

[B61] Qi S. Liu C. Qin M. Tao C. Liu J. Le Y. (2021). An Efficient Photo-Chemo Combination Therapeutic Platform Based on Targeted Reduction-Responsive Self-Crosslinked Polymer Nanocapsules. Mater. Adv. 2, 3020–3030. 10.1039/d1ma00097g

[B62] Qian X. Long L. Shi Z. Liu C. Qiu M. Sheng J. (2014). Star-Branched Amphiphilic PLA-b-PDMAEMA Copolymers for Co-Delivery of miR-21 Inhibitor and Doxorubicin to Treat Glioma. Biomaterials 35, 2322–2335. 10.1016/j.biomaterials.2013.11.039 24332459

[B63] Qian X. Shi Z. Qi H. Zhao M. Huang K. Han D. (2019). A Novel Granzyme B Nanoparticle Delivery System Simulates Immune Cell Functions for Suppression of Solid Tumors. Theranostics 9, 7616–7627. 10.7150/thno.35900 31695790 PMC6831455

[B64] Qian X.-M. Shi Z.-D. Ren Y. Liu C.-Y. Ji Y.-R. Long L.-X. (2013). Synergistic Inhibition of Human Glioma Cell Line by Temozolomide and PAMAM-Mediated miR-21i. J. Appl. Polym. Sci. 127, 570–576. 10.1002/app.37823

[B65] Qin M. Cao Z. Wen J. Yu Q. Liu C. Wang F. (2020). An Antioxidant Enzyme Therapeutic for COVID-19. Adv. Mater. 32, e2004901. 10.1002/adma.202004901 32924219

[B66] Qosa H. Miller D. S. Pasinelli P. Trotti D. (2015). Regulation of ABC Efflux Transporters at Blood-Brain Barrier in Health and Neurological Disorders. Brain Res. 1628, 298–316. 10.1016/j.brainres.2015.07.005 26187753 PMC4681613

[B67] Raghavan R. Brady M. L. Rodríguez-Ponce M. I. Hartlep A. Pedain C. Sampson J. H. (2006). Convection-enhanced Delivery of Therapeutics for Brain Disease, and its Optimization. Neurosurg. Focus 20, E12. 10.3171/foc.2006.20.4.7 16709017

[B68] Ren Y. Wang R. Gao L. Li K. Zhou X. Guo H. (2016). Sequential Co-Delivery of miR-21 Inhibitor Followed by Burst Release Doxorubicin Using NIR-Responsive Hollow Gold Nanoparticle to Enhance Anticancer Efficacy. J. Control. Release 228, 74–86. 10.1016/j.jconrel.2016.03.008 26956593

[B69] Ren Y. Wang R. Liu Y. Guo H. Zhou X. Yuan X. (2014). A Hematoporphyrin-Based Delivery System for Drug Resistance Reversal and Tumor Ablation. Biomaterials 35, 2462–2470. 10.1016/j.biomaterials.2013.12.004 24373420

[B70] René C. A. Parks R. J. (2021). Delivery of Therapeutic Agents to the Central Nervous System and the Promise of Extracellular Vesicles. Pharmaceutics 13 (4), 492. 10.3390/pharmaceutics13040492 33916841 PMC8067091

[B71] Sarin H. (2010). Physiologic Upper Limits of Pore Size of Different Blood Capillary Types and Another Perspective on the Dual Pore Theory of Microvascular Permeability. J. Angiogenes Res. 2, 14. 10.1186/2040-2384-2-14 20701757 PMC2928191

[B72] Shah D. K. Betts A. M. (2013). Antibody Biodistribution Coefficients: Inferring Tissue Concentrations of Monoclonal Antibodies Based on the Plasma Concentrations in Several Preclinical Species and Human. MAbs 5, 297–305. 10.4161/mabs.23684 23406896 PMC3893240

[B73] Sharma A. Liaw K. Sharma R. Spriggs T. Appiani La Rosa S. Kannan S. (2020). Dendrimer-Mediated Targeted Delivery of Rapamycin to Tumor-Associated Macrophages Improves Systemic Treatment of Glioblastoma. Biomacromolecules 21, 5148–5161. 10.1021/acs.biomac.0c01270 33112134

[B74] Sharma A. Subudhi S. K. Blando J. Scutti J. Vence L. Wargo J. (2019). Anti-CTLA-4 Immunotherapy Does Not Deplete FOXP3+ Regulatory T Cells (Tregs) in Human Cancers. Clin. Cancer Res. 25, 1233–1238. 10.1158/1078-0432.CCR-18-0762 30054281 PMC6348141

[B75] Shi Z. D. Qian X. M. Liu C. Y. Han L. Zhang K. L. Chen L. Y. (2013). Aspirin-/TMZ-coloaded Microspheres Exert Synergistic Antiglioma Efficacy via Inhibition of β-catenin Transactivation. CNS Neurosci. Ther. 19, 98–108. 10.1111/cns.12041 23230963 PMC6694282

[B76] Sun P. Xiao Y. Di Q. Ma W. Ma X. Wang Q. (2020). Transferrin Receptor-Targeted PEG-PLA Polymeric Micelles for Chemotherapy Against Glioblastoma Multiforme. Int. J. Nanomed. 15, 6673–6688. 10.2147/IJN.S257459 PMC7494234 32982226

[B77] Tsai Y. C. Vijayaraghavan P. Chiang W. H. Chen H. H. Liu T. I. Shen M. Y. (2018). Targeted Delivery of Functionalized Upconversion Nanoparticles for Externally Triggered Photothermal/Photodynamic Therapies of Brain Glioblastoma. Theranostics 8, 1435–1448. 10.7150/thno.22482 29507632 PMC5835948

[B78] Tu L. Luo Z. Wu Y. L. Huo S. Liang X.-J. (2021). Gold-Based Nanomaterials for the Treatment of Brain Cancer. Cancer Biol. Med. 18 (2), 372–387. 10.20892/j.issn.2095-3941.2020.0524 PMC8185869 34002583

[B79] Vredenburgh J. J. Desjardins A. Herndon J. E. Marcello J. Reardon D. A. Quinn J. A. (2007). Bevacizumab Plus Irinotecan in Recurrent Glioblastoma Multiforme. J. Clin. Oncol. 25, 4722–4729. 10.1200/JCO.2007.12.2440 17947719

[B80] Wang D. Starr R. Chang W. C. Aguilar B. Alizadeh D. Wright S. L. (2020). Chlorotoxin-directed CAR T Cells for Specific and Effective Targeting of Glioblastoma. Sci. Transl Med. 12 (533), eaaw2672. 10.1126/scitranslmed.aaw2672 32132216 PMC7500824

[B81] Wang Q. Ren Y. Mu J. Egilmez N. K. Zhuang X. Deng Z. (2015). Grapefruit-Derived Nanovectors Use an Activated Leukocyte Trafficking Pathway to Deliver Therapeutic Agents to Inflammatory Tumor Sites. Cancer Res. 75, 2520–2529. 10.1158/0008-5472.CAN-14-3095 25883092 PMC4470740

[B82] Wang Q. Zhuang X. Mu J. Deng Z. B. Jiang H. Zhang L. (2013). Delivery of Therapeutic Agents by Nanoparticles Made of Grapefruit-Derived Lipids. Nat. Commun. 4, 1867. 10.1038/ncomms2886 23695661 PMC4396627

[B83] Wen J. Wu D. Qin M. Liu C. Wang L. Xu D. (2019). Sustained Delivery and Molecular Targeting of a Therapeutic Monoclonal Antibody to Metastases in the central Nervous System of Mice. Nat. Biomed. Eng. 3, 706–716. 10.1038/s41551-019-0434-z 31384008 PMC6736720

[B84] Werle M. (2008). Natural and Synthetic Polymers as Inhibitors of Drug Efflux Pumps. Pharm. Res. 25, 500–511. 10.1007/s11095-007-9347-8 17896100 PMC2265773

[B85] Wu D. Qin M. Xu D. Wang L. Liu C. Ren J. (2019a). A Bioinspired Platform for Effective Delivery of Protein Therapeutics to the Central Nervous System. Adv. Mater. 31, e1807557. 10.1002/adma.201807557 30803073 PMC6701476

[B86] Wu D. Yang Y. Xu P. Xu D. Liu Y. Castillo R. (2019b). Real-Time Quantification of Cell Internalization Kinetics by Functionalized Bioluminescent Nanoprobes. Adv. Mater. 31, e1902469. 10.1002/adma.201902469 31402525

[B87] Wu Q. Han J. Li C. Sun T. Xie Z. (2020). Photothermal Therapy Combined with Light-Induced Generation of Alkyl Radicals for Enhanced Efficacy of Tumor Treatment. ACS Appl. Polym. Mater. 2, 4188–4194. 10.1021/acsapm.0c00832

[B88] Wu Y. Fan Q. Zeng F. Zhu J. Chen J. Fan D. (2018). Peptide-Functionalized Nanoinhibitor Restrains Brain Tumor Growth by Abrogating Mesenchymal-Epithelial Transition Factor (MET) Signaling. Nano Lett. 18, 5488–5498. 10.1021/acs.nanolett.8b01879 30067910

[B89] Xu D. Wu D. Qin M. Nih L. R. Liu C. Cao Z. (2019a). Efficient Delivery of Nerve Growth Factors to the Central Nervous System for Neural Regeneration. Adv. Mater. 31, e1900727. 10.1002/adma.201900727 31125138

[B90] Xu J. Yu S. Wang X. Qian Y. Wu W. Zhang S. (2019b). High Affinity of Chlorin e6 to Immunoglobulin G for Intraoperative Fluorescence Image-Guided Cancer Photodynamic and Checkpoint Blockade Therapy. ACS Nano 13, 10242–10260. 10.1021/acsnano.9b03466 31397999

[B91] Ye Z. Zhang T. He W. Jin H. Liu C. Yang Z. (2018). Methotrexate-Loaded Extracellular Vesicles Functionalized with Therapeutic and Targeted Peptides for the Treatment of Glioblastoma Multiforme. ACS Appl. Mater. Inter. 10, 12341–12350. 10.1021/acsami.7b18135 29564886

[B92] Yoo J. Y. Swanner J. Otani Y. Nair M. Park F. Banasavadi-Siddegowda Y. (2019). Oncolytic HSV Therapy Increases Trametinib Access to Brain Tumors and Sensitizes Them *In Vivo* . Neuro Oncol. 21, 1131–1140. 10.1093/neuonc/noz079 31063549 PMC7571492

[B93] Zhan C. Lu W. (2012). The Blood-Brain/Tumor Barriers: Challenges and Chances for Malignant Gliomas Targeted Drug Delivery. Curr. Pharm. Biotechnol. 13, 2380–2387. 10.2174/138920112803341798 23016643

[B94] Zhan Q. Yi K. Qi H. Li S. Li X. Wang Q. (2020). Engineering Blood Exosomes for Tumor-Targeting Efficient Gene/Chemo Combination Therapy. Theranostics 10, 7889–7905. 10.7150/thno.45028 32685027 PMC7359100

[B95] Zhang C. Song J. Lou L. Qi X. Zhao L. Fan B. (2021). Doxorubicin-Loaded Nanoparticle Coated with Endothelial Cells-Derived Exosomes for Immunogenic Chemotherapy of Glioblastoma. Bioeng. Transl. Med. 6, e10203. 10.1002/btm2.10203 34589592 PMC8459598

[B96] Zhang K. Sun X. Zhou X. Han L. Chen L. Shi Z. (2015). Long Non-coding RNA HOTAIR Promotes Glioblastoma Cell Cycle Progression in an EZH2 Dependent Manner. Oncotarget 6, 537–546. 10.18632/oncotarget.2681 25428914 PMC4381613

[B97] Zhao G. Huang Q. Wang F. Zhang X. Hu J. Tan Y. (2018). Targeted shRNA-Loaded Liposome Complex Combined With Focused Ultrasound for Blood Brain Barrier Disruption and Suppressing Glioma Growth. Cancer Lett. 418, 147–158. 10.1016/j.canlet.2018.01.035 29339208

[B98] Zhao M. Van Straten D. Broekman M. L. D. Préat V. Schiffelers R. M. (2020). Nanocarrier-Based Drug Combination Therapy for Glioblastoma. Theranostics 10, 1355–1372. 10.7150/thno.38147 31938069 PMC6956816

[B99] Zheng M. Liu Y. Wang Y. Zhang D. Zou Y. Ruan W. (2019). ROS-responsive Polymeric siRNA Nanomedicine Stabilized by Triple Interactions for the Robust Glioblastoma Combinational RNAi Therapy. Adv. Mater. 31, e1903277. 10.1002/adma.201903277 31348581

[B100] Zhu Y. Jiang Y. Meng F. Deng C. Cheng R. Zhang J. (2018). Highly Efficacious and Specific Anti-Glioma Chemotherapy by Tandem Nanomicelles Co-functionalized With Brain Tumor-Targeting and Cell-Penetrating Peptides. J. Control. Release 278, 1–8. 10.1016/j.jconrel.2018.03.025 29596873

[B101] Zhuang X. Teng Y. Samykutty A. Mu J. Deng Z. Zhang L. (2016). Grapefruit-Derived Nanovectors Delivering Therapeutic miR17 Through an Intranasal Route Inhibit Brain Tumor Progression. Mol. Ther. 24, 96–105. 10.1038/mt.2015.188 26444082 PMC4754550

[B102] Zlokovic B. V. (2008). The Blood-Brain Barrier in Health and Chronic Neurodegenerative Disorders. Neuron 57, 178–201. 10.1016/j.neuron.2008.01.003 18215617

[B103] Zou Y. Sun X. Wang Y. Yan C. Liu Y. Li J. (2020). Single siRNA Nanocapsules for Effective siRNA Brain Delivery and Glioblastoma Treatment. Adv. Mater. 32, e2000416. 10.1002/adma.202000416 32374446

